# Anemia is associated with incidence of dementia: a national health screening study in Korea involving 37,900 persons

**DOI:** 10.1186/s13195-017-0322-2

**Published:** 2017-12-06

**Authors:** Su-Min Jeong, Dong Wook Shin, Ji Eun Lee, Jung Hyeon Hyeon, Jinkook Lee, SangYun Kim

**Affiliations:** 1 Department of Family Medicine, Seoul National University Hospital, Seoul, Republic of Korea; 2 Department of Family Medicine & Supportive Care Center, Samsung Medical Center, Samsung Comprehensive Cancer Hospital, Seoul, Republic of Korea; 3 Department of Economics & Center for Economic & Social Research, University of Southern California, Los Angeles, CA USA; 4 RAND Corporation, Santa Monica, CA USA; 5 Department of Neurology, Seoul National University Bundang Hospital & Seoul National University College of Medicine, Seongnam, Republic of Korea

**Keywords:** Anemia, Dementia, Severity of anemia, ICD-10

## Abstract

**Background:**

The aim of this study was to investigate whether anemia is associated with dementia incidence in the elderly.

**Methods:**

Using the Korean National Health Insurance Service-National Health Screening Cohort (NHIS-HEALS) database, we identified 66-year-old subjects (*n* = 37,900) who were free of dementia and stroke. Anemia (hemoglobin < 12 g/dl for women and < 13 g/dl for men) and the severity of anemia (mild, moderate, or severe) were defined using World Health Organization criteria. The incidence of dementia was identified using International Classification of Diseases, Tenth Revision, dementia diagnosis codes (F00, F01, F02, F03, and G30) with prescription of an antidementia drug. Cox proportional hazards regression models were used to assess HRs for dementia incidence according to anemia.

**Results:**

After adjusting for sex, baseline cognitive state, body mass index, smoking status, household income, disability, depression, hypertension, diabetes, and dyslipidemia, we found a significant association between anemia and dementia incidence (adjusted HR 1.24; 95% CI 1.02–1.51). The adjusted HRs for incidence of dementia according to the severity of anemia were 1.19 (95% CI 0.98–1.45) for those with mild anemia, 1.47 (95% CI 0.97–2.21) for those with moderate anemia, and 5.72 (95% CI 1.84–17.81) for those with severe anemia, showing a significant *p* value for trend (*p* = 0.003).

**Conclusions:**

Anemia is an independent risk factor for dementia incidence, with a marked increase of risk associated with severe anemia.

**Electronic supplementary material:**

The online version of this article (doi:10.1186/s13195-017-0322-2) contains supplementary material, which is available to authorized users.

## Background

The prevalence of dementia is estimated to increase as aging populations continue to advance globally. For people > 60 years of age, dementia was ranked among the leading causes of increased disability-adjusted life-years in 2015 [1]. Considering that patients with dementia represent diverse aspects of deterioration in behavior, cognition, and emotion, they need public and social support as well as informal care provided by their family and other persons [2]. Despite the growing importance of dementia and emerging evidence of modifiable risk factors for reducing the risk of dementia [3], more research on modifiable risk factors for dementia is required. Recent studies have revealed decreasing trends of dementia incidence or prevalence, suggesting that there may be some dementia cases that could be preventable or delayed through managing modifiable risk factors for dementia [4] or improving and increasing educational attainment [5].

Anemia is a common condition in the elderly, with a prevalence > 10% in community-dwelling adults aged ≥ 65 years, and it is increasing with the aging population, according to World Health Organization (WHO) criteria [6]. The treatment of anemia could involve interventions that target its specific causes. One-third of anemia diagnoses in the elderly are attributable to nutritional deficiency, which could easily be treated by adequate nutritional support, such as iron, vitamin B_12_, or folate supplementation [7].

Previous studies have suggested that anemia is a risk factor for cognitive decrease [8–10] and dementia incidence [11–13]. However, some findings have been contradictory [14], and most studies have applied a single binary criterion in defining anemia (e.g., hemoglobin < 13 mg/dl for men and < 12 mg/dl for women) and have not examined possible dose-response relationships [8–13]. Furthermore, there are limitations in studies with a cross-sectional design [10, 12, 15]; in studies with relatively small study populations [8, 9, 11–13]; or related to difficulties in generalizing findings owing to specific populations studied, such as those with chronic kidney disease [14]. Therefore, in the present study, we aimed to evaluate the association between anemia and dementia incidence, considering the severity of anemia.

## Methods

### Study setting

The Korean National Health Insurance (KNHI) program is a mandatory universal public health insurance system that covers the entire Korean population, except for Medicaid beneficiaries in the lowest income bracket (~3% of the population). The KNHI database has been widely used in various epidemiological studies [16, 17] and is described in detail elsewhere [18, 19].

KNHI offers the National Health Screening Program (NHSP) biennially to all members > 40 years of age. This aim of this program is to screen for cardiovascular risk factors, as well as several other pathologic conditions, including anemia. An additional program, the National Screening Program for Transitional Ages (NSPTA), for people of two target ages, 40 years and 66 years, is provided. In the 66-year-old age group, NSPTA includes geriatric, physical, and cognitive functional assessments [20]. Repeat cognitive function tests are then done at two 4-year intervals (at 70 and 74 years of age).

### Study population

This study was conducted using the National Health Insurance Service-National Health Screening Cohort (NHIS-HEALS) database and comprised 515,000 people. This figure represents 10% of a random selection from within the total Korean population, of those who participated in the NHSP at least once in the index year 2002 or 2003, and aged from 40 to 79 years. NHIS-HEALS contains demographic factors such as age, sex, insurance premium (a proxy for economic status) and disability state (categorized into six grades by the National Registration for Disability), results of the NHSP, and information on the use of medical facilities that includes the International Classification of Diseases, Tenth Revision (ICD-10), codes, with prescribed medicines as outpatients, as well as hospitalization.

**Fig. 1:**
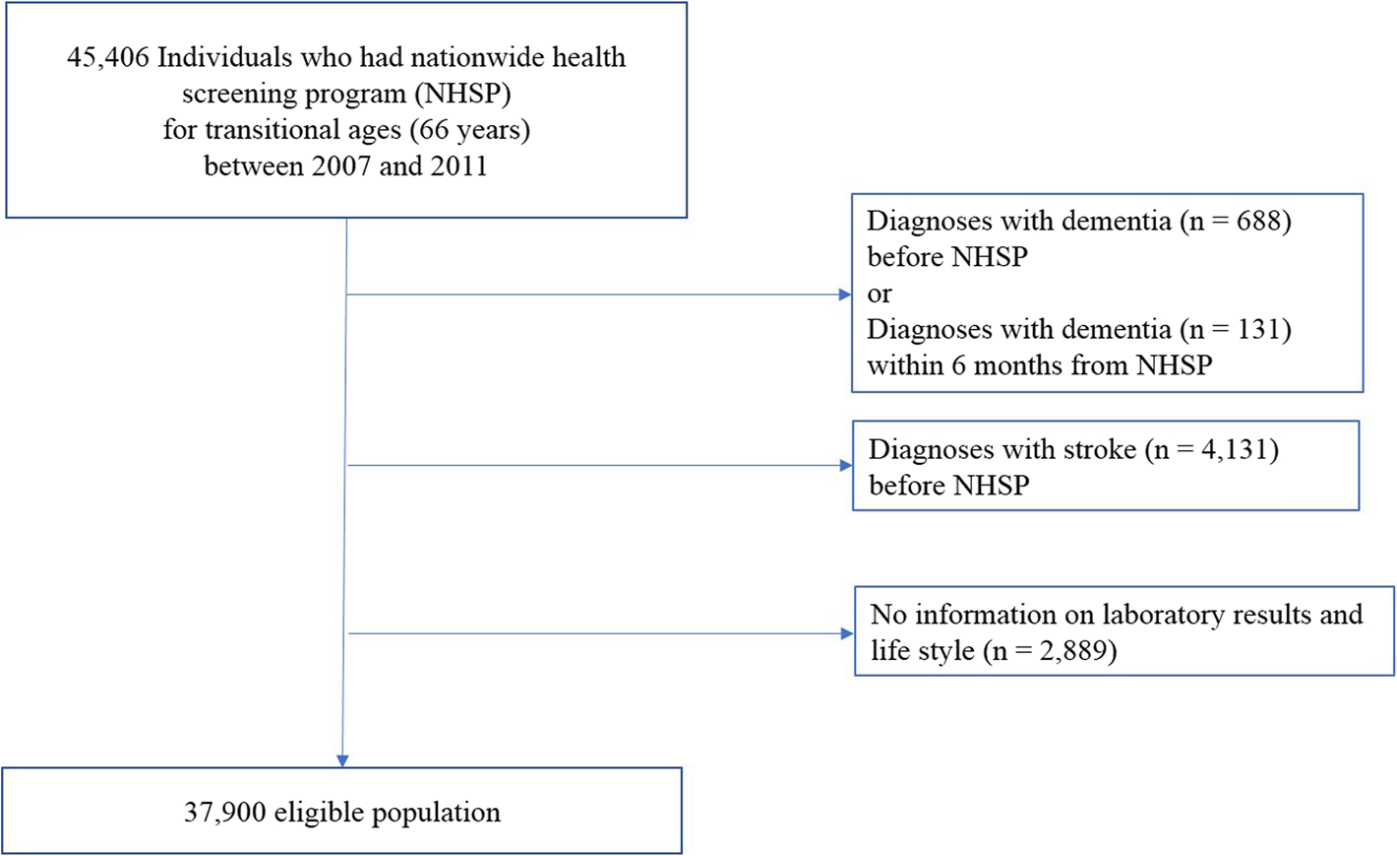
Flowchart of the study population. *NHSP* National Health Screening Program

Within the NHIS-HEALS database, we identified 45,406 eligible individuals who had participated in the NSPTA at age 66, between 2007 and 2011. We excluded individuals who had a diagnosis of dementia before (*n* = 688) or after 6 months (*n* = 131) of the health screening date, using ICD-10 codes of dementia (F00, F01, F02, F03 and G30) taken from KNHI medical service claims data. Moreover, individuals who had stroke-related diagnoses (*n* = 4131) were also excluded (I60, I61, I62, I63 orI64) before the health screening date to eliminate any predisposing factors of vascular dementia. Individuals with information missing regarding baseline laboratory results, life style habits and in the cognitive screening questionnaire (*n* = 2889) were excluded. Finally, a total of 37,900 individuals were included in the analysis (Fig. 1).

### Exposure

Anemia was defined according to WHO criteria as hemoglobin < 12 g/dl for women and < 13 g/dl for men. The severity of anemia was categorized as mild (hemoglobin ≥ 11 g/dl), moderate (hemoglobin 8–10.9 g/dl), or severe (hemoglobin < 8 g/dl), using WHO criteria [21]. Serum hemoglobin concentration was measured using the cyanmethemoglobin method.

### Outcomes

#### Primary outcome

Dementia incidence was determined through the use of prescribed an antidementia drug (donepezil, galantamine, rivastigmine, or memantine) [22], with ICD-10 codes (F00, F01, F02, F03, or G30) used for the first or second diagnosis for medical expense claims submitted to the NHIS until the end of follow-up (31 December 2013) [23, 24]. This definition of dementia incidence was applied to outpatients as well as to hospitalized patients. In Korea, it is required to fulfill KNHI reimbursement criteria to claim expenses. To submit a valid claim for the prescription of an antidementia drug, physicians need to document the evidence for cognitive dysfunction according to relatively strict criteria: (1) Mini Mental State Examination score ≤ 26 and (2) either a Clinical Dementia Rating ≥ 1 or a Global Deterioration Scale score ≥ 3 [25].

#### Secondary outcome

To explain the mechanisms of increased dementia incidence, we also performed subgroup analyses with 11,310 individuals whose cognitive function had been retested at age 70 (2011–2013), 4 years after the starting point (2007–2009). In the NHSP, people are required to complete a cognitive screening questionnaire, the Korean Prescreening Korean Dementia Screening Questionnaire (KDSQ-P), to screen for early dementia at the age of 66 years and every 4 years afterward. The KDSQ-P has shown a significant correlation with the Korean version of the Mini Mental State Examination, with high validity and reliability [26]. Each of five questions can be answered, by self-report or through a caregiver, with three possible answers—“no,” “sometimes yes,” or “frequently yes”—scored as 0, 1, or 2, respectively, with a highest total possible score of 10. Those who have scores ≥ 4 points are expected to have further evaluation for cognitive function assessment. The KDSQ-P score change at 4-year follow-up was also obtained for those with normal cognitive function at baseline (*n* = 8988). Cognitive decline was defined as a transition from a normal KDSQ-P score (< 4) at the age of 66 years (baseline) to an abnormal KDSQ-P score (≥ 4) at the age of 70 years.

### Covariates

Body mass index (BMI) was calculated using weight (kg) divided by height in meters squared (m^2^), and classified as low (< 18.8 kg/m^2^), normal to overweight (18.5–24.9 kg/m^2^), or obese (≥ 25 kg/m^2^). Hypertension, diabetes, and dyslipidemia were defined using physicians’ diagnoses or whether medication was being taken, based on self-reporting. Three questions from the Geriatric Depression Scale relating to loss of interest, feelings of uselessness, and feeling without hope were used to screen for depressive symptoms. If individuals answered affirmatively to any of the three questions, they were defined as having depressive symptoms. The history of smoking status was classified as never smoked, former smoker, or current smoker.

### Statistical analyses

Baseline characteristics of individuals with anemia were compared with those without anemia, using a two-tailed Student’s *t* test for continuous variables and a χ^2^ test for categorical variables. Continuous variables are expressed as mean ± SD. A Kaplan-Meier failure time plot was used to describe the incidence of dementia according to the severity of anemia. Cox regression analysis was performed on factors of dementia incidence according to the presence or degree of anemia. In model 1, dementia incidence was adjusted for sex; baseline KDSQ-P score; and lifestyle and socioeconomic variables, such as BMI, smoking status, household income, and disability. Model 2 was further adjusted for clinical information such as hypertension, diabetes, hypercholesterolemia, and depressive mood. To evaluate an association of anemia with cognitive decline, multivariate logistic regression analyses were used for individuals with normal KDSQ-P scores at baseline, after adjusting for the same covariates as in model 2 in the Cox regression analyses. All statistical analyses were carried out using Stata version 14.1 software (StataCorp, College Station, TX, USA).

## Results

### Baseline characteristics and dementia incidence

The age of the total population in this study was the same, namely 66 years old, and included 18,565 (49%) men (Table 1). The prevalence of anemia at baseline using WHO criteria was 13.3%. Individuals with anemia were more likely to be women, nonsmokers, have a lower BMI, and have a lower household income. Higher systolic blood pressure and higher total cholesterol concentrations were also observed in those without anemia compared with those with anemia. There was no significant difference in baseline KDSQ-P scores between those with anemia and those without anemia (*p* = 0.930). The median follow-up period was 4.4 years, and the median times to dementia incidence were 3.4 years in the nonanemia groups and 3.1 years in the anemia groups. Incidence rates were 5.7 cases per 1000 person-years in the anemia group and 4.4 cases per 1000 person-years in the nonanemia group, respectively. Additionally, most cases of dementia were identified as Alzheimer’s disease (AD)-related dementia (82.5%) when we specified ICD-10 codes of AD (F00 or G30).

**Table 1:** Baseline characteristics of study populations

		Anemia^a^		
	Total^b^	No	Yes	*p* Value
All individuals, *n* (%)	37,900	32,872 (86.7)	5028 (13.3)	
Sex, *n* (%)				< 0.001
Male	18,565 (49.0)	16,583 (89.3)	1982 (10.7)	
Female	19,335 (51.0)	16,289 (84.2)	3046 (15.8)	
Baseline KDSQ-P score^c^, *n* (%)				0.930
< 4	30,967 (81.7)	26,861 (86.7)	4106 (13.3)	
≥ 4	6933 (18.3)	6011 (86.7)	922 (13.3)	
Body mass index, kg/m^2^, *n* (%)				< 0.001
< 18.5	831 (2.2)	609 (73.3)	222 (26.7)	
18.5–24.9	23,074 (60.9)	19,775 (85.7)	3299 (14.3)	
≥ 25	12,995 (36.9)	12,488 (89.2)	1507 (10.8)	
Smoking status, *n* (%)				< 0.001
Never	26,722 (70.5)	22,864 (85.6)	3858 (14.4)	
Former	6310 (16.7)	5626 (89.2)	684 (10.8)	
Current	4868 (12.8)	4382 (90.0)	486 (10.0)	
Household income status percentiles, *n* (%)				0.001
≤ 20 (low)	6972 (18.4)	5956 (85.4)	1016 (14.6)	
30–50	8491 (22.4)	7376 (86.9)	1115 (13.1)	
60–80	12,153 (32.1)	10,529 (86.6)	1624 (13.4)	
≥ 90 (high)	10,284 (27.1)	9011 (87.6)	1273 (12.4)	
Disability, *n* (%)				0.471
No	37,839 (99.8)	32,821 (86.7)	5018 (13.3)	
Yes	61 (0.2)	51 (83.6)	10 (16.4)	
Depressive symptoms, *n* (%)				< 0.001
No	28,804 (76.0)	25,081 (87.1)	3723 (12.9)	
Yes	9096 (24.0)	7791 (85.7)	1305 (14.3)	
Hypertension, *n* (%)				0.015
No	22,017 (58.1)	19,017 (86.4)	3000 (13.6)	
Yes	15,883 (41.9)	13,855 (87.2)	2028 (12.8)	
Diabetes, *n* (%)				0.002
No	27,638 (72.9)	24,064 (87.1)	3574 (12.9)	
Yes	10,262 (27.1)	8808 (85.8)	1454 (14.2)	
Dyslipidemia, *n* (%)				0.141
No	29,195 (77.0)	25,281 (86.6)	3914 (13.4)	
Yes	8705 (23.0)	7591 (87.2)	1114 (12.8)	
SBP, mmHg mean (SD)	128.8 (15.7)	129.1 (15.6)	126.9 (15.8)	< 0.001
FBG, mg/dl, mean (SD)	102.1 (25.1)	102.2 (24.9)	101.5 (26.5)	0.070
TC, mg/dl, mean (SD)	198.7 (38.1)	200.0 (37.7)	190.1 (39.2)	< 0.001

*Abbreviations: KDSQ-P* Prescreening Korean Dementia Screening Questionnaire, *SBP* systolic blood pressure, *FBG* fasting blood glucose, *TC* total cholesterol

^a^According to World Health Organization anemia criteria, anemia was defined as hemoglobin < 13 g/dl for men and < 12 g/dl for women

^b^Column percentage

^c^KDSQ-P score ≥ 4 needs further evaluation for cognitive function

### Anemia and risk of dementia

Individuals with anemia showed a higher incidence of dementia compared to those without anemia (Table 2). The HR was 1.32 and the 95% CI for those with anemia was 1.09–1.60. In the adjusted models, those with anemia had a significant association with dementia incidence in model 1 (adjusted HR = 1.25; 95% CI 1.03–1.52) and in model 2 (adjusted HR = 1.24; 95% CI 1.02–1.51). Being women, having an abnormal KDSQ-P score at baseline, with lower household income and an absence of dyslipidemia were associated with a higher incidence of dementia.

**Table 2:** HRs for the incidence of dementia using baseline anemia data

		aHR (95% CI)	
	Unadjusted HR	Model 1^a^	Model 2^a^
Anemia^b^	1.32 (1.09–1.60)	1.25 (1.03–1.52)	1.24 (1.02–1.51)
Female sex		1.61 (1.32–1.96)	1.60 (1.32–1.94)
Baseline KDSQ-P^c^ score ≥ 4		1.72 (1.47–2.01)	1.68 (1.43–1.97)
BMI, kg/m^2^			
< 18.5		0.96 (0.58–1.58)	0.95 (0.57–1.56)
18.5–24.9		1.00	1.00
≥ 25		0.94 (0.80–1.09)	0.93 (0.80–1.09)
Smoking status			
Never		1.00	1.00
Former		1.04 (0.80–1.36)	1.03 (0.79–1.35)
Current		1.17 (0.90–1.53)	1.16 (0.89–1.52)
Household income status percentiles, *n* (%)			
≤ 20 (low)		1.00	1.00
30–50		0.95 (0.76–1.18)	0.95 (0.76–1.18)
60–80		0.88 (0.71–1.08)	0.88 (0.72–1.08)
≥ 90 (high)		0.77 (0.62–0.95)	0.77 (0.62–0.9)
Disability		2.25 (0.72–7.01)	2.29 (0.74–7.13)
Depressive symptoms			1.15 (0.73–1.35)
Hypertension			1.02 (0.87–1.19)
Diabetes			1.18 (0.96–1.45)
Dyslipidemia			0.70 (0.57–0.87)

*Abbreviations: BMI* Body mass index, *KDSQ-P* Korean Dementia Screening Questionnaire-P, *aHR* Adjusted HR

^a^Adjusted for sex, baseline KDSQ-P score, BMI, smoking status, household income, and disability (model 1) and for the same variables plus depressive symptoms, hypertension, diabetes, and dyslipidemia (model 2)

^b^According to World Health Organization anemia criteria, anemia was defined as hemoglobin < 13 g/dl for men and < 12 g/dl for women.

^c^KDSQ-P score ≥ 4 needs further evaluation for cognitive function

**Fig. 2:**
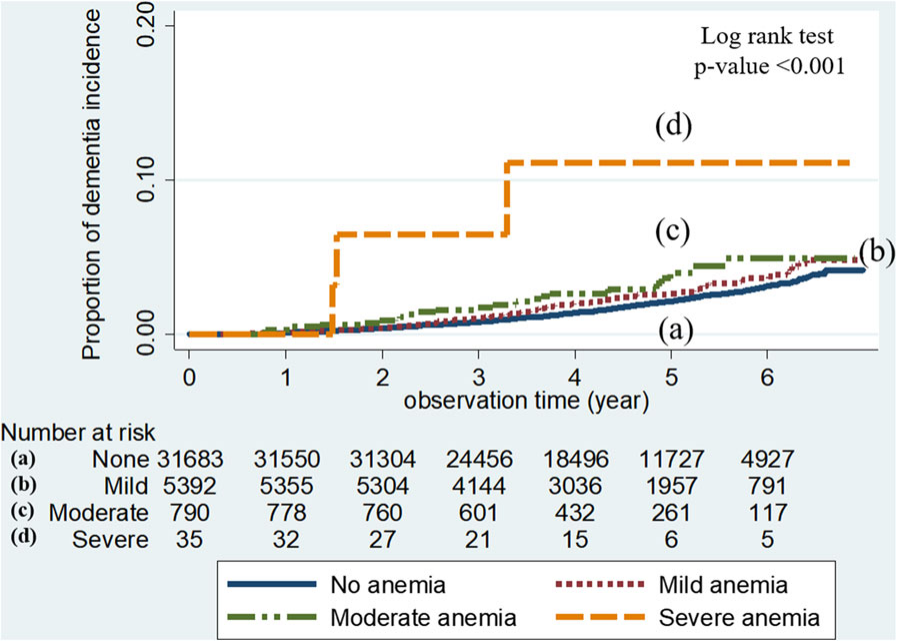
Kaplan-Meier survival analysis for incidence of dementia according to the severity of anemia. The severity of anemia was categorized as (*a*) none (hemoglobin ≥ 13 g/dl in men and hemoglobin ≥ 12 g/dl in women), (*b*) mild (hemoglobin ≥ 11 g/dl), (*c*) moderate (hemoglobin 8–10.9 g/dl), or (*d*) severe (hemoglobin < 8 g/dl) according to World Health Organization criteria. The incidence of dementia increased according to the severity of anemia, with a significant *p* value for trend (< 0.001)

Analyses considering the severity of anemia showed that there was a significant trend of association between hemoglobin level and risk of dementia (*p* < 0.05 in all models). The adjusted HRs for incidence of dementia according to the severity of anemia were 1.19 (95% CI 0.98–1.45) in mild anemia and 1.47 (95% CI 0.97–2.21) in moderate anemia (Table 3). The severe anemia group had an especially significant association with high risk for dementia incidence (adjusted HR 5.72; 95% CI 1.84–17.81) (Fig. 2). Furthermore, when we adjusted for chronic kidney disease in addition to model 2 among 37,937 subjects who had information on serum creatinine levels, severe anemia was consistently associated with the incidence of dementia (*see* Additional file 1).

**Table 3:** HRs for incidence of dementia according to severity of anemia

				aHR^a^ (95% CI)	
Severity of anemia^b^	Total patients	Dementia, *n* (%)	Unadjusted HR	Model 1	Model 2
None	31,683	594 (1.87)	1.00	1.00	1.00
Mild	5392	126 (2.34)	1.27 (1.05–1.54)	1.20 (0.99–1.46)	1.19 (0.98–1.45)
Moderate	790	24 (3.04)	1.68 (1.12–2.53)	1.48 (0.98–2.24)	1.47 (0.97–2.21)
Severe	35	3 (8.57)	5.99 (1.92–18.61)	5.65 (1.82–18.59)	5.72 (1.84–17.81)
*p* Value for trend			< 0.001	0.003	0.003

*aHR* Adjusted HR

^a^Adjusted for sex, baseline Prescreening Korean Dementia Screening Questionnaire score, body mass index, smoking status, household income, disability (model 1) and for the same variables plus depressive symptoms, hypertension, diabetes, and dyslipidemia (model 2)

^b^The severity of anemia was classified as mild (≥ 11 g/dl), moderate (8–10.9 g/dl), or severe (< 8 g/dl) according to World Health Organization criteria

### Anemia and cognitive decline

There was a significant association between anemia and cognitive decline after 4-year follow-up for individuals with a normal KDSQ-P score (*n* = 8988) (adjusted OR 1.30; 95% CI 1.03–1.62) (Table 4).

**Table 4:** Association between anemia and new cognitive decline after 4-year follow-up among individuals with normal cognitive function at baseline (*n* = 8988)

	Unadjusted OR (95% CI)	aOR^a^ (95% CI)
Anemia^b^	1.32 (1.09–1.60)	1.30 (1.03–1.62)
Female sex		1.73 (1.39–2.16)
BMI, kg/m^2^		
< 18.5		0.86 (0.47–1.56)
18.5–24.9		1.00
≥ 25		1.04 (0.88–1.23)
Smoking status		
Never		1.00
Former		1.29 (0.97–1.71)
Current		1.37 (1.02–1.83)
Household income status percentiles		
≤ 20 (low)		1.00
30–50		1.18 (0.91–1.53)
60–80		1.17 (0.91–1.49)
≥ 90 (high)		1.12 (0.87–1.44)
Disability		2.88 (0.62–13.30)
Depressive symptoms		1.33 (1.11–1.60)
Hypertension		0.98 (0.82–1.17)
Diabetes		0.98 (0.78–1.23)
Dyslipidemia		0.92 (0.73–1.16)

*aOR* Adjusted odds ratio, *BMI* Body mass index

^a^Adjusted for sex, baseline Prescreening Korean Dementia Screening Questionnaire score, BMI, smoking status, household income, disability, depressive symptoms, hypertension, diabetes, and dyslipidemia

^b^According to World Health Organization anemia criteria, anemia was defined as hemoglobin < 13 g/dl for men and < 12 g/dl for women

## Discussion

Using an exceptionally large cohort dataset of elderly people, we confirmed that anemia is associated with the incidence of dementia. In addition, for the first time, to the best of our knowledge, we found that severe anemia (hemoglobin < 8 g/dl) was independently associated with a fivefold higher dementia incidence than in those without anemia. Our results, derived from subgroup data, also showed that anemia was significantly associated with cognitive decline after a 4-year follow-up, providing further support for the association of anemia and dementia incidence.

Many previous studies linking anemia and dementia have suggested various mechanisms, unfortunately none of which are fully established, including the following:

1. Chronic brain hypoxia related to anemia may partially contribute to cognitive function decline through accelerating the accumulation of amyloid-β [27].
2. Anemia has shown an association with the progression of white matter hyperintensity [28] and cerebral cortical atrophy [29].
3. Iron deficiency in the brain may affect its neurotransmitter metabolism and function through interference with rate-limiting enzymes that are dependent on iron [30].
4. Vitamin B_12_ or folate deficiency is recognized as a risk factor for dementia, which may be based on altering homocysteine and acetylcholine metabolism [31].

However, depressive symptoms were more prevalent in those with anemia in this study. Depressive symptom could act as a factor common to both anemia and dementia. Anemia could be a consequence of malnutrition linked to loss of appetite, an important depressive symptom [32], and late-life depression was independently associated with dementia [33].

Our study has a particular strength in that we showed a dose-response relationship of anemia severity and dementia risk. Our large study population (*N* = 37,900 compared with 2552 in the Health, Aging and Body Composition [ABC] study and 13,133 in the Atherosclerosis Risk in Communities study [ARIC]) enabled us to secure enough statistical power for analysis of the data by severity, and the dose-response relationship revealed by our study provides further support for the association of anemia and dementia risk. One possible mechanism supporting the dose-response relationship is that mild to moderate anemia may have less effect on oxygen delivery to the brain through a compensatory reaction, such as vascular dilation to maintain cerebral blood flow, compared with severe anemia, which may exceed the threshold of compensation [34, 35]. An association of anemia with a decline of cognitive function in cognitively intact elderly persons at baseline further suggests that anemia could be an etiological risk factor for dementia rather than representing a co-occurrence of the two factors through another shared risk factor. Cognitive decline precedes dementia, and elucidation of this relationship is likely to shed light on the possible mechanisms between anemia and dementia.

The clinical importance of our study lies in the fact that anemia is a largely correctable disease. Especially in low-income and middle-income countries, most anemia arises from an inadequate nutritional supply (iron or vitamins) [36], and anemia resulting from chronic disease might be corrected through treatment (for example, erythropoietin therapy for chronic kidney disease). If this is the case, then dementia may be at least partially preventable through treating anemia.

There are several limitations of our study. First, because our data are based on routinely collected health screening and claims data, and because this study was not a prospective cohort study designed for studying dementia specifically, we did not have all pertinent information relevant to a dementia study. We did not have genetic information, such as *APOE4* carrier status, and we were not able to assess education and literacy levels, which might affect cognitive function [37]. However, we could adjust for baseline cognitive function, minimizing the risk of influence from those other relevant characteristics. A second potential limitation is that the incidence of dementia was not regularly followed using formal cognitive testing, and there is a possibility that people with dementia had been not diagnosed and treated and therefore were not filing claims for prescriptions. However, under the KNHI system, access to healthcare and the use of antidementia drugs are high in Korea, and there is no strong reason to believe that there is a difference in such health-seeking behavior due to the presence of anemia. Third, we could not distinguish the subtypes of dementia, because we thought that the subtypes of dementia could not be clearly verified using claims data, sometimes in actual practice. However, there was a similar trend when we performed analysis restricted to those who have ICD-10 code diagnoses only for AD (F00 and G30) with drug prescription (*see* Additional file 2). Fourth, cautious interpretation is needed in that only 35 cases of severe anemia among 39,700 subjects were associated with incidence of dementia compared with the nonanemia group. There might be a reverse causality between anemia and incidence of dementia, even though we excluded subjects who had diagnosed dementia 6 months before and after the health screening date.

## Conclusions

We found that anemia is an independent risk factor for the incidence of dementia, especially when it is severe. Future studies should elucidate the precise mechanism of association between anemia and the incidence of dementia, as well as whether interventions designed to address anemia are effective in reducing dementia risk.

## Additional files

Additional file 1: Table S1 Association between anemia and incidence of dementia after additionally adjusting for chronic kidney disease in 37,397 subjects. (DOCX 16 kb)

Additional file 2: Table S2 HRs for Alzheimer’s disease according to the severity of anemia. (DOCX 13 kb)

## Abbreviations

ABC study: Health, Aging and Body Composition study
AD: Alzheimer’s disease
aHR: Adjusted HR
aOR: Adjusted OR
ARIC: Atherosclerosis Risk in Communities study
BMI: Body mass index
DBP: Diastolic blood pressure
FBG: Fasting blood glucose
ICD-10: International Classification of Diseases, Tenth Revision
KDSQ-P: Prescreening Korean Dementia Screening Questionnaire
KNHI: Korean National Health Insurance
NHIS-HEALS: National Health Insurance Service-National Health Screening Cohort
NHSP: National Health Screening Program
NSPTA: National Screening Program for Transitional Ages
SBP: Systolic blood pressure
TC: Total cholesterol
WHO: World Health Organization
