# Supplementary material for: Anemia is associated with incidence of dementia: a national health screening study in Korea involving 37,900 persons
Source: Alzheimers Res Ther. 2017 Dec 6;9:94. doi: 10.1186/s13195-017-0322-2 (PMC5719530; doi:10.1186/s13195-017-0322-2)
Supplement: Supplementary file 1 — Association between anemia and incidence of dementia after additionally adjusting for chronic kidney disease in 37,397 subjects. (DOCX 16 kb) [file 13195_2017_322_MOESM1_ESM.docx]

**Additional table 1.** Association between anemia and incidence of dementia after additionally adjusting for chronic kidney disease in 37,397 subjects

|  |  |  | Total  N (%) | Dementia  N (%) | aHR^a^  (95% CI) |
| --- | --- | --- | --- | --- | --- |
| Anemia | |  | 37,397 |  | 1.24 (1.02 – 1.51) |
| None | |  | 31,273 (83.6) | 576 (1.8) | 1.00 |
| Mild | |  | 5,319 (14.2) | 122 (2.3) | 1.19 (0.97 – 1.44) |
| Moderate | |  | 771 (2.1) | 23 (3.0) | 1.44 (0.94 – 2.18) |
| Severe | |  | 34 (0.1) | 3 (8.8) | 5.98 (1.92 – 18.6) |
| Chronic kidney disease^b^ | |  | 4,270 (11.4) | 107 (2.5) | 1.26 (1.02 – 1.55) |

^a^ Adjusted for sex, baseline KDSQ-P score, BMI, smoking status, household income, disability, depressive symptoms, hypertension, diabetes, dyslipidemia and chronic kidney disease

^b^ Chronic kidney disease was defined as estimated glomerular filtration rate (eGFR) of less than 60 mL/min/1.73 m^2^.

aHR adjusted hazard ratio
