# Supplementary material for: Anemia is associated with incidence of dementia: a national health screening study in Korea involving 37,900 persons
Source: Alzheimers Res Ther. 2017 Dec 6;9:94. doi: 10.1186/s13195-017-0322-2 (PMC5719530; doi:10.1186/s13195-017-0322-2)
Supplement: Supplementary file 2 — HRs for Alzheimer’s disease according to the severity of anemia. (DOCX 13 kb) [file 13195_2017_322_MOESM2_ESM.docx]

**Additional table 2.** Hazard ratios for Alzheimer's disease ^a^ according to the severity of anemia

| Severity of Anemia |  | Total  N | Dementia  N (%) |  | aHR^b^ (95% CI) |
| --- | --- | --- | --- | --- | --- |
| None | | 31,683 | 492 (1.55) |  | 1.00 |
| Mild | | 5,392 | 104 (1.93) |  | 1.15 (0.93 – 1.42) |
| Moderate | | 790 | 17 (2.15) |  | 1.14 (0.70 – 1.85) |
| Severe | | 35 | 3 (8.57) |  | 6.57 (2.11 – 20.5) |
| *p*-for trend | |  |  |  | 0.069 |

^a^ Alzheimer’s disease was defined as ICD-codes (F00 and G30) with new prescription of antidementia drug

^b^ Adjusted for sex, baseline KDSQ-P score, BMI, smoking status, household income, disability, depressive symptoms, hypertension, diabetes and dyslipidemia

aHR adjusted hazard ratio
